# The role of bacterial skin infections in atopic dermatitis: expert statement and review from the International Eczema Council Skin Infection Group

**DOI:** 10.1111/bjd.18643

**Published:** 2019-12-04

**Authors:** H. Alexander, A.S. Paller, C. Traidl‐Hoffmann, L.A. Beck, A. De Benedetto, S. Dhar, G. Girolomoni, A.D. Irvine, P. Spuls, J. Su, J.P. Thyssen, C. Vestergaard, T. Werfel, A. Wollenberg, M. Deleuran, C. Flohr

**Affiliations:** ^1^ Unit for Population‐Based Dermatology Research St John's Institute of Dermatology Guy's and St Thomas’ NHS Foundation Trust and King's College London London SE1 7EH U.K.; ^2^ Departments of Dermatology and Pediatrics Northwestern University Feinberg School of Medicine Chicago IL U.S.A.; ^3^ Chair and Institute of Environmental Medicine UNIKA‐T Technical University of Munich and Helmholtz Zentrum München Augsburg Germany; ^4^ CK‐CARE Christine Kühne Center for Allergy Research and Education Davos Switzerland; ^5^ Department of Dermatology University of Rochester Medical Center Rochester NY U.S.A.; ^6^ Department of Dermatology College of Medicine University of Florida Gainesville FL U.S.A.; ^7^ Department of Pediatric Dermatology Institute of Child Health Kolkata India; ^8^ Department of Medicine Section of Dermatology and Venereology University of Verona Verona Italy; ^9^ Department of Clinical Medicine Trinity College Dublin Dublin Ireland; ^10^ Dermatology Children's Health Ireland Dublin Ireland; ^11^ National Children's Research Centre Dublin Ireland; ^12^ Department of Dermatology Amsterdam Public Health, Infection and Immunity Amsterdam UMC University of Amsterdam Amsterdam the Netherlands; ^13^ Departments of Dermatology and Paediatrics Murdoch Children's Research Institute University of Melbourne and Monash University Eastern Health Melbourne VIC Australia; ^14^ Department of Dermatology and Allergy Herlev‐Gentofte Hospital Hellerup Denmark; ^15^ Department of Dermatology Aarhus University Hospital Aarhus Denmark; ^16^ Department of Dermatology and Allergy Hannover Medical School Hannover Germany; ^17^ Department of Dermatology and Allergology Ludwig Maximilian University Munich Germany

## Abstract

Patients with atopic dermatitis (AD) have an increased risk of bacterial skin infections, which cause significant morbidity and, if untreated, may become systemic. *Staphylococcus aureus* colonizes the skin of most patients with AD and is the most common organism to cause infections. Overt bacterial infection is easily recognized by the appearance of weeping lesions, honey‐coloured crusts and pustules. However, the wide variability in clinical presentation of bacterial infection in AD and the inherent features of AD – cutaneous erythema and warmth, oozing associated with oedema, and regional lymphadenopathy – overlap with those of infection, making clinical diagnosis challenging. Furthermore, some features may be masked because of anatomical site‐ and skin‐type‐specific features, and the high frequency of *S. aureus* colonization in AD makes positive skin swab culture of suspected infection unreliable as a diagnostic tool. The host mechanisms and microbial virulence factors that underlie *S. aureus* colonization and infection in AD are incompletely understood. The aim of this article is to present the latest evidence from animal and human studies, including recent microbiome research, to define the clinical features of bacterial infections in AD, and to summarize our current understanding of the host and bacterial factors that influence microbial colonization and virulence.

Patients with atopic dermatitis (AD; also known as ‘atopic eczema’) have an increased risk of recurrent skin infections.^1^, ^2^, ^3^, ^4^ *Staphylococcus aureus* is the most common infectious organism, although beta‐haemolytic streptococci may also be involved.^5^, ^6^, ^7^, ^8^

The mechanisms underlying bacterial infection in AD are multifactorial and include both host and bacterial factors. The reduced skin barrier, cutaneous innate and adaptive immune abnormalities and trauma from scratching all contribute to the increased risk of skin infection.^9^, ^10^, ^11^, ^12^, ^13^ The host skin microbiota may play a role in protecting against *S. aureus* colonization and infection in patients with AD.^14^, ^15^, ^16^, ^17^ Bacterial virulence factors, such as the superantigens, proteases and cytolytic phenol‐soluble modulins (PSMs) secreted by *S. aureus*, cause skin inflammation and may also contribute to bacterial persistence and/or epithelial penetration and infection.^12^, ^18^, ^19^

The complex interaction between bacteria and host results in wide variability in the clinical presentation of infection in AD and can make the diagnosis challenging. Cutaneous infection may be associated with concomitant AD flares, and the classic signs of infection (erythema, oozing and crusting and increased cutaneous warmth) are masked by similar clinical features of AD itself. Increases in erythema in individuals with darker skin types are more difficult to appreciate, making diagnosis yet more challenging. Pustules are an uncommon sign of bacterial infection in AD, but if present they can allow the diagnosis to be made with greater certainty. Diagnosis and management decisions are further complicated by the fact that the main causative organism, *S. aureus*, commonly colonizes even nonlesional, clinically unaffected AD skin, thus limiting the usefulness of bacterial cultures in identifying the causative organism.

Untreated bacterial skin infection in AD may become systemic and lead to life‐threatening complications including sepsis, endocarditis and bone and joint infections.^20^, ^21^, ^22^ Despite the significant morbidity caused by bacterial skin infection in AD, there is a lack of consensus on how to define and treat associated bacterial colonization and infection. Although there are many diagnostic criteria for AD itself, there are no validated diagnostic criteria for infected AD.^23^

The International Eczema Council, a group of approximately 100 experts in AD worldwide, has recently initiated a taskforce to define the role of bacterial skin infections and their management in AD through consensus statements in an effort to provide level D evidence. It is hoped that input from clinical experts will contribute to better defining the wide‐ranging clinical presentations of *S. aureus* infection in AD and, more importantly, to identify better those who may benefit from existing or novel antimicrobial treatments. Based on a systematic search of the literature, including terms for AD and ‘infection’, ‘bacteria’, ‘staphylococcus aureus’ and ‘microbiome’ (detailed search strategy available on request), this narrative review defines the clinical features of bacterial infection in AD and our current understanding of the host and bacterial factors that influence microbial colonization and virulence.

## Clinical features of bacterial skin infection in atopic dermatitis

**Figure 1:**
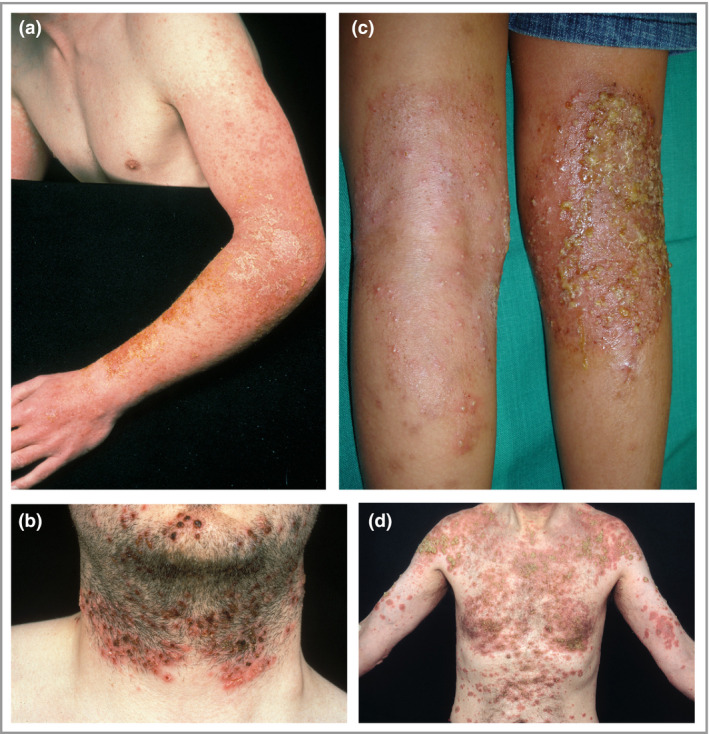
Clinical features of bacterial skin infection in atopic dermatitis. Clinical features of *S. aureus* infection in atopic dermatitis lesions include (a) weeping, honey‐coloured crusts; (b) folliculitis; and (c) pustulation. (d) Beta‐haemolytic streptococcal infection may present with well‐defined bright red erythema.

The typical clinical signs of overt bacterial skin infection in AD are well recognized. More specific signs of *S. aureus* infection in AD lesions include weeping, honey‐coloured crusts, and pustules, both interfollicular and follicular based (folliculitis) (Fig. 1a, b).^6^, ^24^ Pustules are an uncommon feature of infection in AD, but may be associated with significant pruritus and even pain (Fig. 1c).^25^ By contrast, beta‐haemolytic streptococcal infection may present with well‐defined, bright red erythema, thick‐walled pustules and heavy crusting (Fig. 1d).^7^, ^26^ In severe cases, cutaneous bacterial infection may cause abscesses – especially with methicillin‐resistant *S. aureus* (MRSA) infection – fever and lymphadenopathy. A complication in diagnosing infection in AD is the common association with a disease flare. Features of flared AD (increased erythema, oedema, papulation, oozing and excoriation) can mask and/or resemble signs of infection.

### Concomitant viral infection

**Figure 2:**
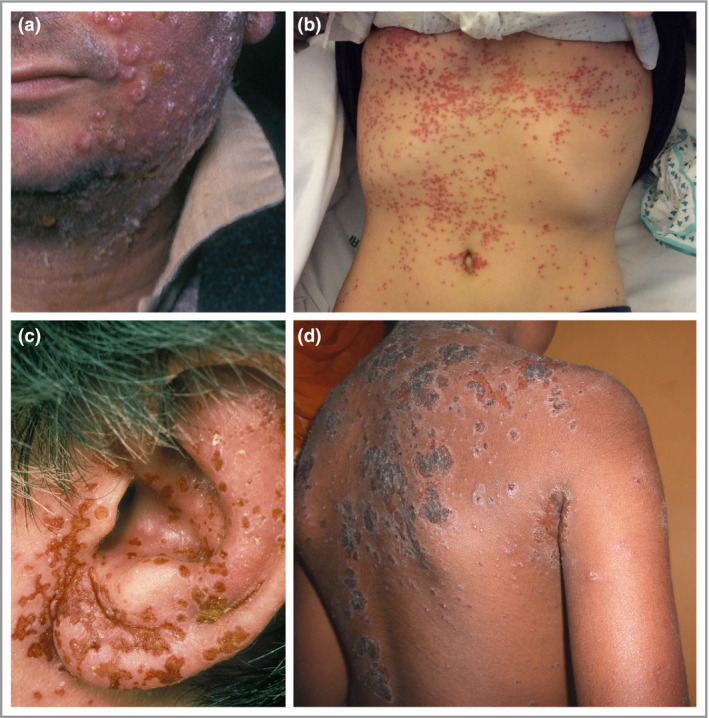
Clinical features of eczema herpeticum. (a, b) Early eczema herpeticum lesions are superficial clusters of dome‐shaped vesicles and/or small, round, punched‐out erosions. (c, d) As the disease progresses, the lesions commonly become superficially infected with *Staphylococcus aureus* and may have the characteristic impetiginized scale.

Several nonbacterial infections can occur concomitantly with bacterial skin infection and can resemble bacterial infections, requiring consideration in the differential diagnosis. For instance, eczema herpeticum (EH) is caused by the local spread of herpes simplex virus, which favours AD lesional skin and is commonly observed in the context of an AD flare.^27^ Early in the course of EH the characteristic skin lesions are superficial clusters of dome‐shaped vesicles and/or small, round, punched‐out erosions (Fig. 2a, b).^27^ As the disease progresses, lesions may become superficially infected with *S. aureus* and may develop an impetiginized scale (Fig. 2c, d).^12^ EH typically arises in involved AD skin, most frequently on the face, neck, upper trunk and antecubital/popliteal areas with AD, and is often accompanied by fever, malaise and lymphadenopathy.^28^, ^29^ Moderate‐to‐severe AD, filaggrin loss‐of‐function mutation, a history of *S. aureus* skin infection, greater allergen sensitization and type 2 immunity are important risk factors for EH.^30^, ^31^, ^32^ Staphylococcal α‐toxin and reductions in the tight junction protein claudin‐1 result in greater epidermal spread of herpes simplex virus *in vitro*.^33^, ^34^ This infection can spread rapidly and, in severe cases, may lead to keratoconjunctivitis and encephalitis.

### Concomitant fungal colonization

**Figure 3:**
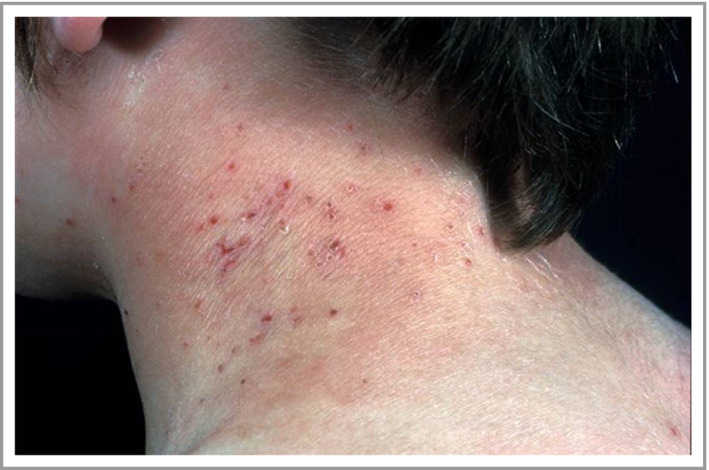
*Malassezia* colonization in atopic dermatitis, which may drive inflammation in patients who have head and neck dermatitis.

Fungal colonization can also complicate the clinical picture of AD. For instance, *Malassezia* colonization is thought to drive inflammation in AD in a subset of patients who typically have dermatitis in areas with a high density of sebaceous glands (e.g. head, neck, and upper chest and back) (Fig. 3). This seborrhoeic distribution overlaps with, but is distinct from, the distribution of allergic contact dermatitis or airborne allergy, which typically involve the upper face, eyelids and periorbital regions, anterior neck, postauricular area and exposed areas on the arms. *Malassezia* is a commensal yeast. Although it is not more abundant on AD skin,^35^ patients with AD are more frequently sensitized to *Malassezia*.^36^, ^37^, ^38^ In some patients, sensitization to yeast antigens induces autoreactivity to human proteins via molecular mimicry, leading to sustained skin inflammation.^39^, ^40^ Cross‐reactivity between *Malassezia*‐specific IgE and *Candida albicans* has also been shown.^41^ A systematic review of the eight published randomized controlled trials evaluating the benefit of antifungal therapy found that five trials demonstrated a benefit from antifungal drugs and three trials found no benefit compared with placebo or standard therapy.^38^

## Bacterial skin infection in different ethnic skin types

**Figure 4:**
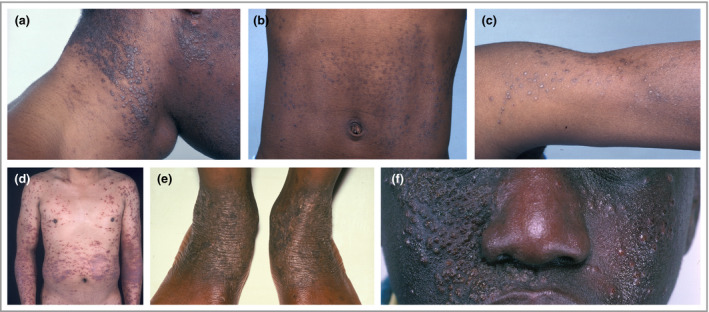
Atopic dermatitis in different ethnic skin types. In dark‐skinned individuals perifollicular accentuation is often present in atopic dermatitis, and erythema appears violaceous.

There is wide variation in the clinical manifestation of AD in different ethnic groups. This may be a result of underlying genetic variation, which influences AD susceptibility and clinical presentation, inadequate early intervention because of masking of erythema in dark skin, and differences in both treatment response and environmental exposures.^42^ In dark‐skinned individuals, perifollicular accentuation is often present and erythema appears violaceous and often muted (Fig. 4).^43^, ^44^, ^45^ This can lead to poor recognition of inflammation, underestimation of disease severity and inadequate intervention. Patients with AD of African descent often have extensor disease rather than the characteristic flexural lesions.^45^ Importantly, *S. aureus* strain differences, including variability in the presence of superantigen genes, has been shown between European American, African American and Mexican American patients with AD.^46^

## Methicillin‐resistant *Staphylococcus aureus*

**Figure 5:**
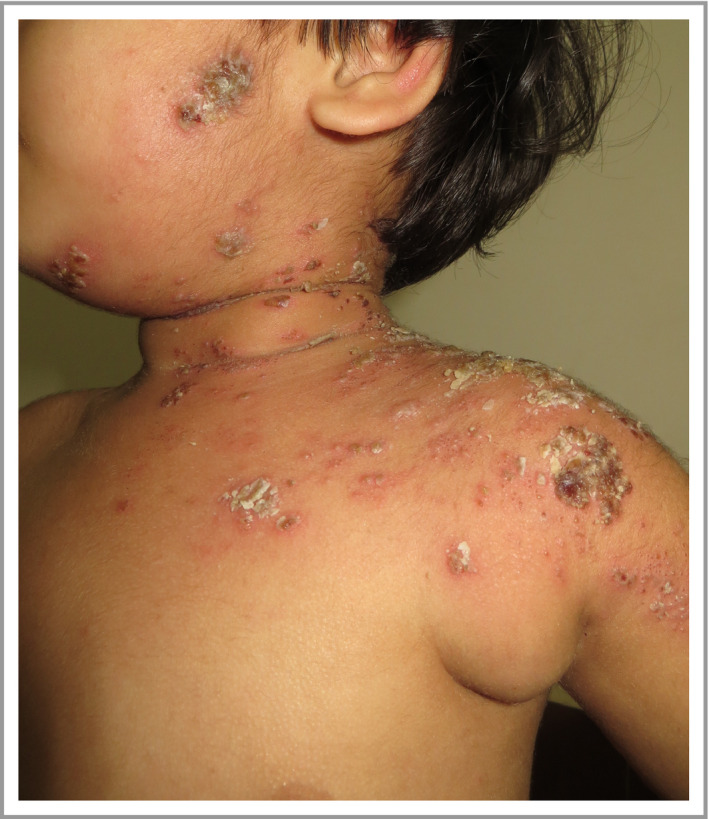
Methicillin‐resistant *Staphylococcus aureus* infection in atopic dermatitis may cause recurrent flares that are resistant to standard treatment regimens.

As in healthy people who are colonized by MRSA, patients with AD often have recurrent infections and disease flares that are resistant to standard treatment regimens (Fig. 5). The prevalence of MRSA skin colonization varies significantly with geographical location and study setting in both healthy and diseased populations. It is therefore difficult to compare accurately the prevalence of MRSA colonization between AD and healthy cohorts. For example, in the U.S.A. there is significant state‐wide variation, with the rate of MRSA colonization varying between 0·3% and 13% in people with AD.^3^, ^47^, ^48^, ^49^ In another study, 4–19% of children with AD from the U.K. and Ireland were found to be colonized with MRSA.^50^, ^51^ The reported prevalence of MRSA colonization in patients with AD in Sri Lanka is 8%, and in Korea 3–14%.^52^, ^53^, ^54^, ^55^ A meta‐analysis of MRSA colonization in the general population reported a prevalence of 0·2–7% worldwide.^56^ The authors describe significant study heterogeneity. In a subgroup analysis that excluded people with prior healthcare contact, the prevalence of MRSA colonization was found to be very low (0·2%).

Although some studies suggest that MRSA colonization rates are higher in people with AD than in the general population, other studies have found much lower rates. For instance, a cross‐sectional study of 200 patients with AD in Canada found MRSA in only one individual.^57^ Similarly, children with AD from San Diego were found to have a lower rate of community‐acquired MRSA colonization than the general outpatient paediatric population.^58^ Further research is needed to understand the significance of MRSA in AD.

## Staphylococcus aureus colonization in atopic dermatitis

Most patients with AD are colonized by *S. aureus*. A recent meta‐analysis found that the pooled prevalence of *S. aureus* colonization of lesional AD skin is 70%, of nonlesional AD skin 39% and of the nares 62%.^59^ However, the prevalence varies greatly across studies, from 22% to 99% in lesional skin and 3% to 79% in nonlesional skin.^59^, ^60^, ^61^, ^62^, ^63^ Most patients colonized by *S. aureus* do not exhibit overt signs of infection, and 10% of healthy individuals carry *S. aureus*.^62^, ^64^

*Staphylococcus aureus* colonization can be associated with three main clinical scenarios in AD: (i) stable or baseline AD without clinical evidence of overt infection; (ii) AD flare without clinical evidence of overt infection; and (iii) overtly infected AD with the classical symptoms as described above. Although antimicrobial therapy is clearly essential for patients with overtly infected AD, the clinical significance, recognition and management of *S. aureus* colonization without clinical evidence of infectious disease are not fully understood. Some studies show that patients with AD improve with topical and systemic antibiotic treatments, even without overt signs of secondary infection.^65^, ^66^, ^67^, ^68^, ^69^, ^70^ However, other studies have found no clinical benefit of antibiotic treatment over corticosteroid therapy alone.^63^, ^71^ A 2010 Cochrane review found no support for routine topical or systemic antistaphylococcal interventions in AD that is not clinically infected, although the studies were generally short term and of poor quality.^72^

It is likely that the density of *S. aureus* is more relevant than simply the presence of the bacteria. The density of *S. aureus* colonization correlates with the severity of AD.^73^, ^74^, ^75^, ^76^ Williamson and Kligman used an early method of quantitative bacteriology to compare the effects of topical and systemic antibiotics on *S. aureus* in AD.^77^ The detergent scrub technique was used on AD lesions to obtain bacterial samples, which were incubated before the *S. aureus* density was measured. They found that appreciable clinical improvement with antibiotic therapy occurred only in patients whose AD lesions were infected by *S. aureus* at a density of greater than 10^6^ colony‐forming units per cm^2^.^61^, ^68^ Similarly, microbiome studies of paediatric patients with AD show that the relative abundance of *S. aureus* is associated with disease flares and correlates with severity.^78^, ^79^, ^80^, ^81^

In addition to bacterial abundance, there are several additional factors that determine whether *S. aureus* successfully colonizes the skin in AD and whether this results in clinically relevant infection. Casadevall and Pirofski described the ‘damage–response framework’ approach to microbial pathogenesis.^82^, ^83^ The basic tenets of this concept are that host and microbe interact to create a spectrum of possible states, ranging from commensalism and colonization to disease. Disease results from damage to the host, which can come from the host response, the microbe or both. The damage–response framework defines infection as the acquisition of a microbe, but it does not necessarily mean the microbe is causing disease. Infection results in disease when the host–microbe interaction produces sufficient damage to become clinically apparent.^84^

**Figure 6:**
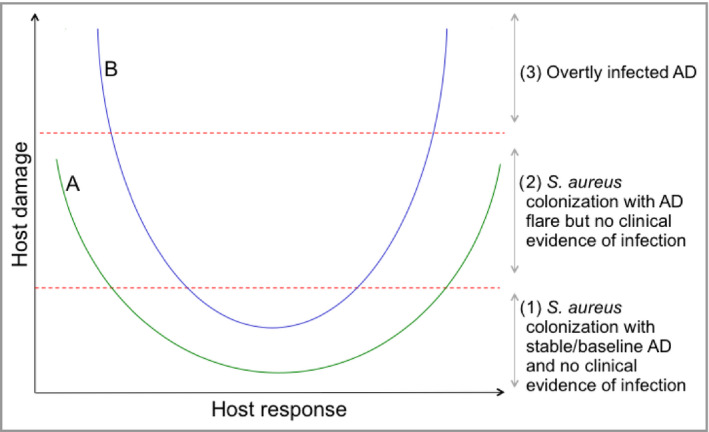
Hypothetical damage–response framework for *Staphylococcus aureus* in atopic dermatitis (AD).^82^ Different host–*S. aureus* interactions result in different damage–response relationships. Curves A and B represent the damage–response relationships of *S. aureus* with two different hosts or those of a single host with two different *S. aureus* strains. The outcome for the host depends on the strength of the host response to *S. aureus* or the virulence of *S. aureus*. During intermediate host responses neither interaction (A or B) causes clinical evidence of infection, as the amount of damage incurred by the host is insufficient (1). However, in the setting of weak or strong responses both interactions cause an AD flare (2) and interaction B causes overtly infected AD (3). The position of the curve is determined by multiple host and *S. aureus* factors.

This approach is a framework that advances thinking beyond the classic microbe‐centric Koch's postulates that dominated microbiological thought for more than a century. It may be a useful approach for understanding the *S. aureus*–host interaction in AD and the range of clinical scenarios that can arise (Fig. 6). We have some understanding of the various bacterial and host factors that contribute during *S. aureus* infection in AD. However, the key questions to be answered are (i) which of these factors lead to worsening inflammation in AD? and (ii) can a threshold of host damage resulting from the *S. aureus*–host interaction be defined, beyond which antibiotics prove beneficial? If the key host and microbial factors that determine these outcomes are identified, then targeting of these specific factors with novel immunotherapies or selective antimicrobial therapies may become a reality.^14^, ^85^

## Host factors associated with *Staphylococcus aureus* colonization

**Figure 7:**
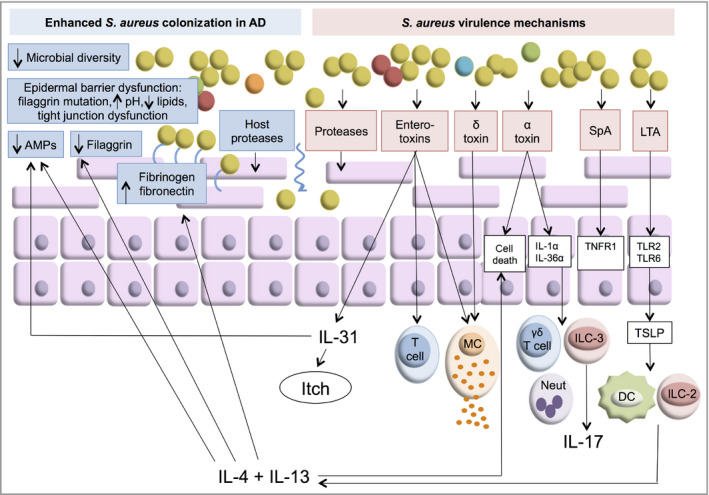
Possible mechanisms of *Staphylococcus aureus* colonization and virulence in atopic dermatitis (AD). *Staphylococcus aureus* colonization is increased in AD skin. This may be due to epidermal barrier dysfunction, reduced levels of antimicrobial peptides (AMPs), reduced microbial diversity or increased fibrinogen and fibronectin. Proteases produced by the host and *S. aureus* allow the bacteria to penetrate into the deeper layers of the skin. Staphylococcal enterotoxins (SEs) stimulate polyclonal T‐cell responses, SE‐specific IgE responses and interleukin (IL)‐31 expression. α‐Toxin can cause keratinocyte death and can activate keratinocyte IL‐1α and IL‐36α production to stimulate γδT cells, innate lymphoid cell (ILC)‐3‐mediated IL‐17 release and neutrophil (Neut) recruitment. δ‐Toxin causes mast cell (MC) degranulation. Staphylococcal protein A (SpA) activates proinflammatory pathways via tumour necrosis factor receptor 1 (TNFR1) on keratinocytes. *Staphylococcus aureus* lipoteichoic acid (LTA) and lipoproteins activate Toll‐like receptor (TLR)2 and TLR6 to produce thymic stromal lymphopoietin (TSLP), which activates dendritic cells (DC) and ILC‐2, leading to production of T helper cell (Th)2 cytokines.

Adults with AD who are colonized with *S. aureus* have more severe disease, and greater T helper type 2 (Th2) immune deviation, allergen sensitization and barrier dysfunction than noncolonized patients with AD.^86^ Some studies have found that filaggrin mutations are associated with *S. aureus* colonization in AD, but others have not.^86^, ^87^, ^88^ The increased susceptibility to *S. aureus* colonization and infection in AD is multifactorial and driven by both skin barrier abnormalities and innate and adaptive immune responses (Fig. 7).

### The impaired skin barrier

The impaired skin barrier in AD is characterized by reduced very‐long‐chain epidermal lipids, defective tight junctions, differentiation in protein deficiency (including from filaggrin loss‐of‐function mutations), enhanced protease activity and increased skin‐surface pH. This impaired barrier provides a favourable environment for *S. aureus* colonization.^89^, ^90^, ^91^, ^92^ The deposition of stratum corneum (SC) fibronectin, to which *S. aureus* adheres, is increased in AD.^26^, ^93^, ^94^ *Staphylococcus aureus* clumping factor B binds to loricrin and cytokeratin 10 and promotes adhesion of *S. aureus* to the stratum corneum in AD.^95^ Antimicrobial peptides (AMPs) such as β‐defensins and cathelicidins are also reduced in AD lesions.^96^

### Type 2 inflammation

Type 2 inflammatory pathways, in which the cytokines interleukin (IL)‐4 and IL‐13 play a major role, drive inflammation in AD. Th2 cytokines reduce expression of important skin barrier proteins: filaggrin, loricrin and involucrin.^97^, ^98^ The expression of fibronectin is increased by IL‐4 and may facilitate *S. aureus* adherence in AD.^99^ The failure to mount an appropriate AMP response in AD may also be due to the suppressive effects of IL‐4 and IL‐13, and may enhance *S. aureus* colonization further.^12^, ^13^, ^100^

A recent pooled analysis of seven randomized, placebo‐controlled dupilumab trials in adults with moderate‐to‐severe AD found that bacterial skin infections were significantly less common in the dupilumab groups than in the placebo group.^101^ Similarly, a meta‐analysis of data from eight dupilumab trials found that patients treated with dupilumab had a lower risk of skin infection than those treated with placebo.^102^ The reduced rate of skin infection with dupilumab supports the role of a Th2‐driven host skin barrier defect in infection in AD, which after treatment may become a less favourable environment for bacteria. This shift may be mediated by inhibition of type 2 inflammatory cytokines, reduced scratching, or microbiome changes induced by dupilumab. Dupilumab treatment results in increased microbial diversity and decreased *S. aureus* abundance in AD.^103^

### The skin microbiome

Microbial diversity is reduced in AD and inversely correlates with disease severity.^78^, ^79^, ^81^ Skin commensal microbes, including coagulase‐negative staphylococci (CoNS), may aid skin homeostasis and provide protection against *S. aureus*. Thus, the diminution of commensal skin microbiota with flares may promote *S. aureus* colonization and infection in AD. During flares of paediatric AD, both *Staphylococcus epidermidis* and *S. aureus* are increased, suggesting a compensatory role for *S. epidermidis*.^78^ This skin commensal promotes AMP expression by cultured keratinocytes via Toll‐like receptor 2 signalling.^104^ Furthermore, *S. epidermidis* produces PSMγ and PSMδ, which enhance AMP effects and inhibit growth of *S. aureus* and group A *Streptococcus in vitro*.^105^ Cutaneous application of antimicrobial CoNS strains to adults with AD decreased colonization by *S. aureus* within 24 h of a single application.^14^

In addition to inhibiting *S. aureus* colonization, CoNS also reduce *S. aureus*‐driven skin inflammation. CoNS from healthy skin produce autoinducing peptides that inhibit the *S. aureus* accessory gene regulatory quorum sensing system, leading to reduced expression of the *S. aureus* virulence factor PSMα *in vitro* and reduced *S. aureus*‐induced skin barrier damage in mice.^16^ *Cutibacterium acnes* supresses growth of MRSA in mouse skin through glycerol fermentation, leading to short‐chain fatty acid production and reduced bacterial intracellular pH.^15^ Treatment with the Gram‐negative *Roseomonas mucosa*, collected from healthy human skin, inhibits the growth of *S. aureus in vitro* and results in reduced inner‐ear thickness in a mouse model of AD.^106^ In human studies, spraying *R. mucosa* onto lesional AD skin of the antecubital area improved AD severity and reduced the need for topical corticosteroids.^17^ MRSA colonization is associated with reduced microbial diversity compared with methicillin‐sensitive *S. aureus* colonization of AD lesional skin and greater decreases in the relative abundance of skin commensal bacteria, including *Cutibacterium*,* Streptococcus* and *Corynebacterium*.^47^ Further research is needed to understand the interactions between *S. aureus* and commensal organisms, and how these organisms relate to host immune responses.

## *Staphylococcus aureus* factors promoting colonization and virulence

*Staphylococcus aureus* exacerbates AD by secreting virulence factors that affect the epidermis (leading to inflammation and skin barrier disruption) and factors that hamper innate and adaptive immune responses (Fig. 7). Staphylococcal superantigens activate polyclonal T‐cell responses without prior antigen processing and by activating epithelial cells via CD40.^107^, ^108^, ^109^ Several of the staphylococcal enterotoxins can also act as allergens to stimulate staphylococcal exotoxin‐specific IgE production.^110^ Staphylococcal enterotoxin B increases the expression of IL‐31, which is well known to cause pruritus in AD.^111^ IL‐31 also suppresses filaggrin and AMP expression, resulting in increased *S. aureus* colonization.^112^, ^113^ Superantigen‐producing strains are found in over 80% of *S. aureus* isolates from patients with AD.^114^ MRSA produces higher levels of superantigen enterotoxins than methicillin‐sensitive *S. aureus*.^115^

Additional toxins such as the staphylococcal PSMs, including δ‐toxin and α‐toxin, may additionally enhance the virulence of *S. aureus* in AD. The δ‐toxin is a potent inducer of mast cell degranulation *in vitro* and in mouse models of AD.^116^ α‐Toxin treatment of AD skin causes keratinocyte death, which is enhanced by IL‐4 and IL‐13.^117^ Recent studies have shown that α‐toxin activates keratinocyte IL‐1α and IL‐36α production, which stimulates γδT cells, innate lymphoid cell (ILC)‐3‐mediated IL‐17 release and neutrophil recruitment.^118^, ^119^ Filaggrin protects keratinocytes by mediating the secretion of sphingomyelinase, an enzyme that reduces the number of α‐toxin binding sites on the keratinocyte surface.^120^ *Staphylococcus aureus* growth and virulence factor production are reduced in the presence of filaggrin breakdown products.^121^ These studies suggest that *S. aureus*‐produced mediators potentiate the effects of *S. aureus* in AD, and filaggrin‐deficient epidermis may be particularly susceptible to *S. aureus*.

Staphylococcal protein A activates proinflammatory pathways via tumour necrosis factor receptor 1 on keratinocytes.^122^ *Staphylococcus aureus* lipoteichoic acid and lipoproteins activate Toll‐like receptors 2 and 6 to exacerbate AD and stimulate release of thymic stromal lymphopoietin (TSLP) from keratinocytes. TSLP activates dendritic cells and ILC‐2, leading to further production of type 2 cytokines.^12^, ^123^ *Staphylococcus aureus* proteases are required for penetration of the bacteria into the deeper layers of the skin and the induction of Th2 cytokine production.^124^ *Staphylococcus aureus* also stimulates keratinocytes to increase their endogenous protease activity.^125^ Whole‐genome sequencing of *S. aureus* has recently revealed higher levels of antimicrobial resistance genes in *S. aureus* isolates from children with AD than in those from healthy control children, suggesting additional potential *S. aureus* virulence mechanisms in AD.^52^, ^126^

## Conclusions

Bacterial infection in AD is common and causes significant morbidity. Overt bacterial infection is easily recognized. However, less overt manifestation of infection may be more difficult to diagnose, especially given the greater risk of infection with flares (themselves associated with increased erythema and oozing), as well as the limited value of culture, given the high rates of colonization. Although we have some understanding of how *S. aureus* colonizes the skin and causes inflammation in AD, many questions related to this complex relationship remain unanswered. Further research is needed for better definition of features that distinguish infection from colonization. Future work of the International Eczema Council, through expert consensus statements, aims to provide guidance regarding the practical use of antimicrobial therapy in atopic dermatitis. Improving our understanding of *S. aureus* virulence mechanisms and downstream host immune mediators of *S. aureus*‐driven inflammatory pathways may help to identify novel therapeutic targets for infection in AD.

## Appendix

Conflicts of interest: A.S.P. is an investigator for AbbVie, AnaptysBio, Castle Creek, Eli Lilly, Galderma, Incyte, Janssen, LEO, Novartis and Regeneron; and a consultant for AbbVie, Amgen, Asana, Castle Creek, Dermavant, Dermira, Galderma, Eli Lilly, Forte, LEO, Matrisys, Menlo, Morphosys/Galapagos, Novartis, Patagonia, Pfizer, Pierre Fabre, Regeneron, Sanofi Genzyme and UCB. L.A.B. is an investigator for AbbVie, Eli Lilly, LEO Pharma, Pfizer and Regeneron; and a consultant for AbbVie, Allakos, Arena Pharma, AstraZeneca, Connect Biopharma, Incyte, LEO Pharma, Lilly, Novan, Novartis, Pfizer, Regeneron, Sanofi and UCB; and has stock in Pfizer and Medtronics. S.D. is a key opinion leader and member of the scientific advisory boards of Galderma India, Sun Pharma, ALKEM Pharma, Curatio Health Care, BIOCON Pharma and Sanofi India. G.G. has been principal investigator in clinical trials sponsored by and/or and has received personal fees from AbbVie, Abiogen, Almirall, Amgen, Biogen, Celgene, Eli Lilly, Genzyme, LEO Pharma, Menlo Therapeutics, Novartis, Pfizer, Regeneron, Samsung, Sandoz and Sanofi. A.D.I. is a coinvestigator of the U.K. National Institute for Health Research‐funded TREAT trial (ISRCTN15837754) and the U.K.–Irish Atopic Eczema Systemic Therapy Register (A‐STAR; ISRCTN11210918). He is a principal investigator in the European Union Horizon 2020‐funded BIOMAP Consortium (http://www.biomap-imi.eu). He has received consulting fees from AbbVie, Genentech, Janssen, LEO Pharma, Novartis, Regeneron, Sanofi Genzyme and Pfizer. P.S. has consulted in the past for Sanofi (11 October 2017) and AbbVie (4 December 2017) (unpaid) and is involved in performing clinical trials with many pharmaceutical companies that manufacture drugs used for the treatment of conditions including psoriasis and atopic dermatitis, for which financial compensation is paid to the department. J.S. has been an investigator, consultant or advisor for AbbVie, Amgen, Eli Lilly, Janssen/JNJ, Meda, Novartis, Pfizer, Pierre Fabre and Sanofi. J.P.T. is an advisor for Sanofi‐Genzyme, Pfizer, AbbVie, LEO Pharma and Eli Lilly & Co; and an investigator for AbbVie, LEO Pharma, Eli Lilly & Co and Sanofi‐Genzyme, presently for Sanofi‐Genzyme, LEO Pharma and Regeneron. C.V. is an investigator and lecturer for LEO Pharma, Sanofi‐Genzyme, AbbVie, Galapagos, Novartis and Pfizer. T.W., as principal investigator of the German TREAT registry on atopic dermatitis, has received honoraria for invited talks or scientific advice and research grants from AbbVie, Almirall, ALK Scherax, Astellas, Janssen/JNJ, LEO, Lilly, Meda, Novartis, Pfizer, Regeneron/Sanofi, Roche, Stallergen, Takeda and Ziarco. A.W. has been an investigator, advisor or lecturer for AbbVie, Almirall, Chugai, Eli Lilly, Galapagos, Galderma, LEO Pharma, MedImmune, Novartis, Pfizer, Pierre Fabre, Regeneron and Sanofi‐Aventis. M.D. is a consultant, investigator, scientific advisory board member and/or lecturer for AbbVie, Eli Lilly, Galapagos, LEO Pharma, Novartis, Pfizer, La Roche‐Posay, Regeneron Pharmaceuticals, Sanofi‐Genzyme, Almirall and Pierre Fabre. C.F. is chief investigator of the U.K. NIHR‐funded TREAT and SOFTER trials, and A‐STAR. He is also a principal investigator in the European Union Horizon 2020‐funded BIOMAP Consortium. His department has also received funding from Sanofi‐Genzyme for skin microbiome work.
